# Supplementary figures and images for: Learning the statistics and landscape of somatic mutation-induced insertions and deletions in antibodies
Source: PLoS Comput Biol. 2022 Jun 2;18(6):e1010167. doi: 10.1371/journal.pcbi.1010167 (PMC9197026; doi:10.1371/journal.pcbi.1010167)

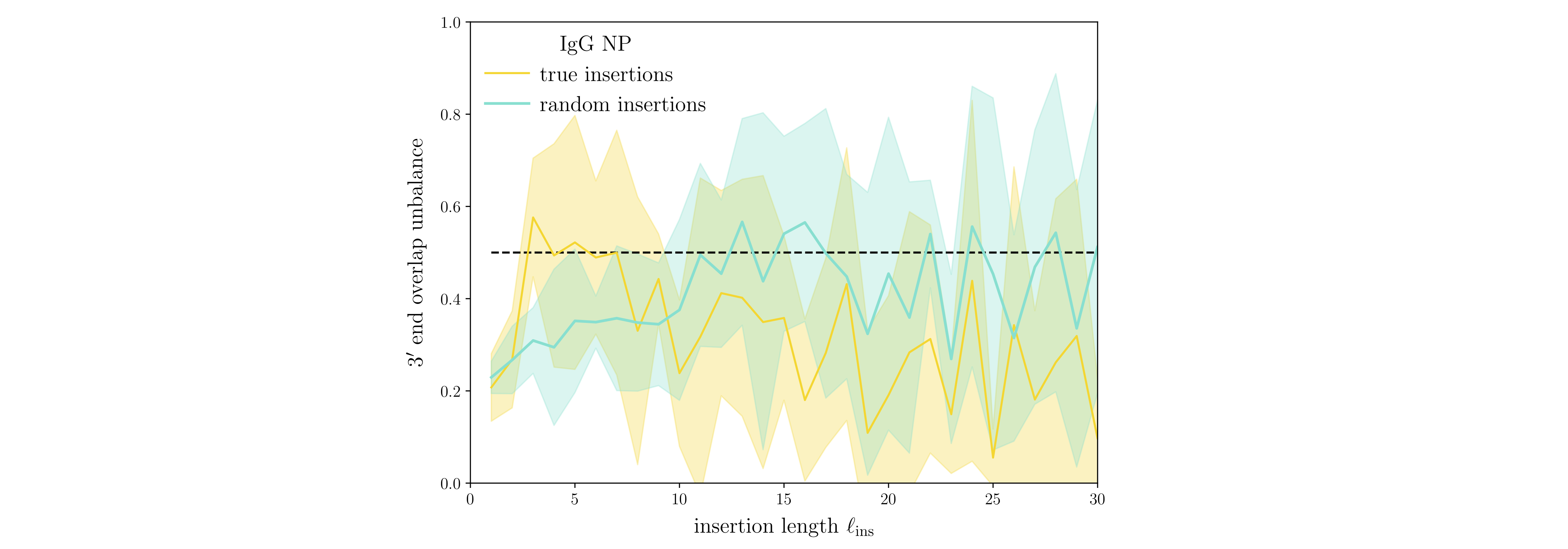

Supplement: S1 Fig — For comparison, overlap between random insertions and flanking regions is also reported (details in the main text). A weak preference for the 5′ end is supported by a KS-test p-value of 0.04, when comparing true insertions with randomized ones. (TIFF) [file pcbi.1010167.s001.tiff]

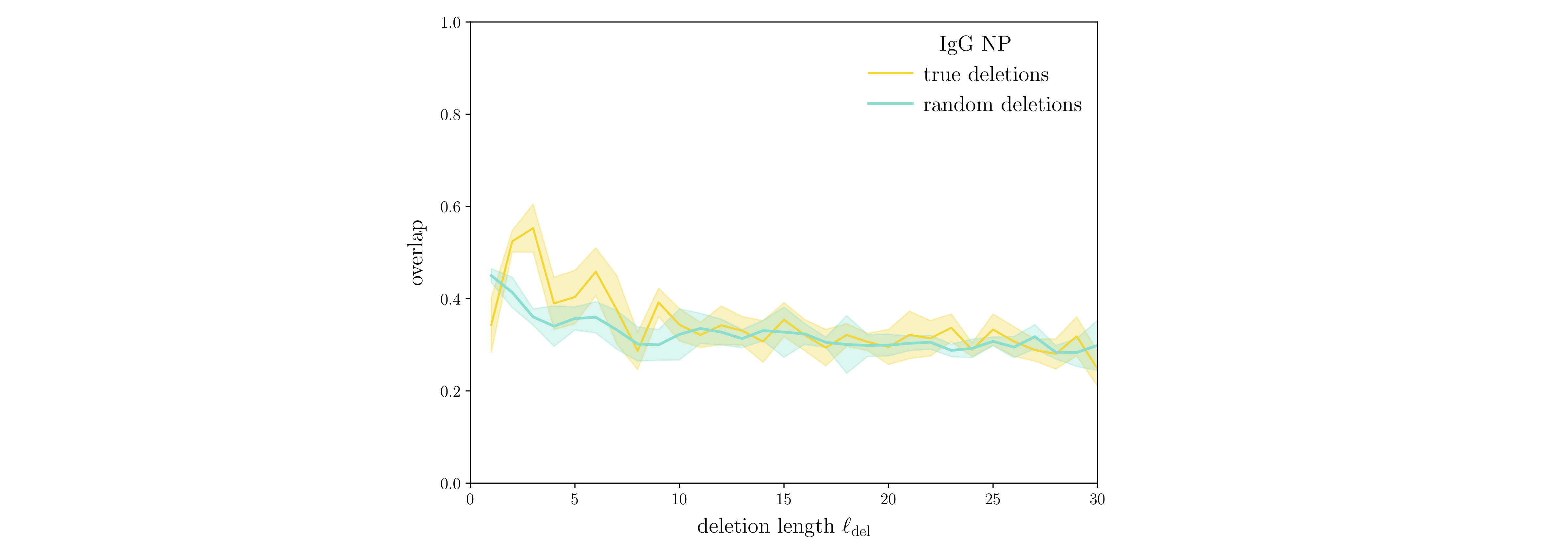

Supplement: S2 Fig — For comparison, overlap between random deletions and flanking regions is also reported (details in the main text). (TIFF) [file pcbi.1010167.s002.tiff]

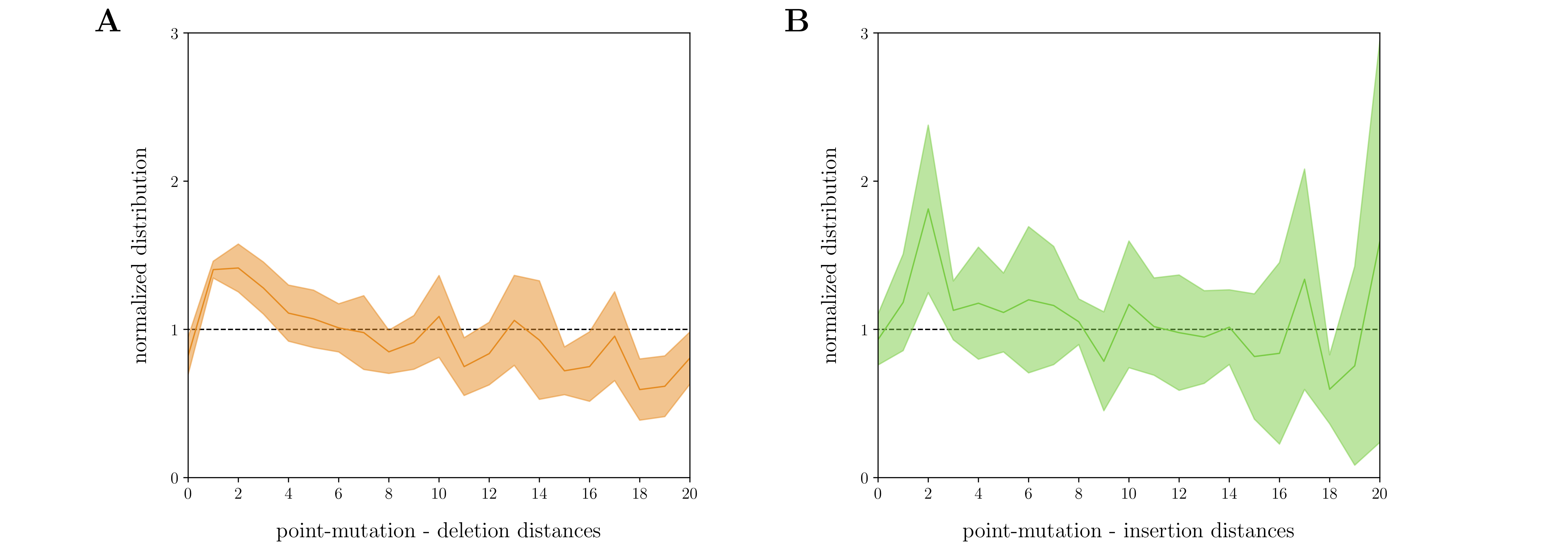

Supplement: S3 Fig — Distribution of the distance (in base pairs) separating (A) deletions or (B) insertions from the closest point mutation in nonproductive IgG sequences, normalized by the null expectation obtained by reshuffling indel and point mutations between sequences with the same V gene (to control for the positional biases of Fig 2). Average and error bars are over the 9 donors. (TIFF) [file pcbi.1010167.s003.tiff]

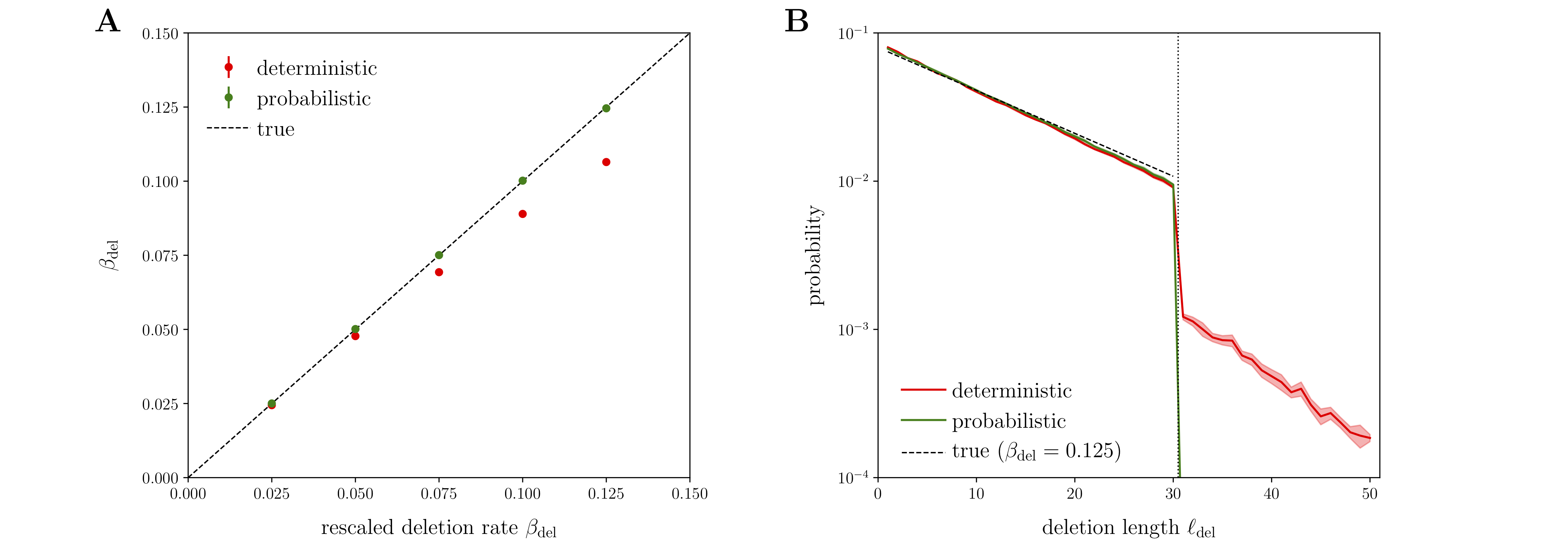

Supplement: S4 Fig — (A) Probabilistic vs deterministic estimates of the rescaled deletion rate βdel at increasing values of indels density. Averages values plus one standard deviation error bars are obtained over the N independent synthetic repertoires. (B) Deletion length profiles for the largest value of βdel considered in panel (A); mean and standard deviations over the N independent realizations. (TIFF) [file pcbi.1010167.s004.tiff]

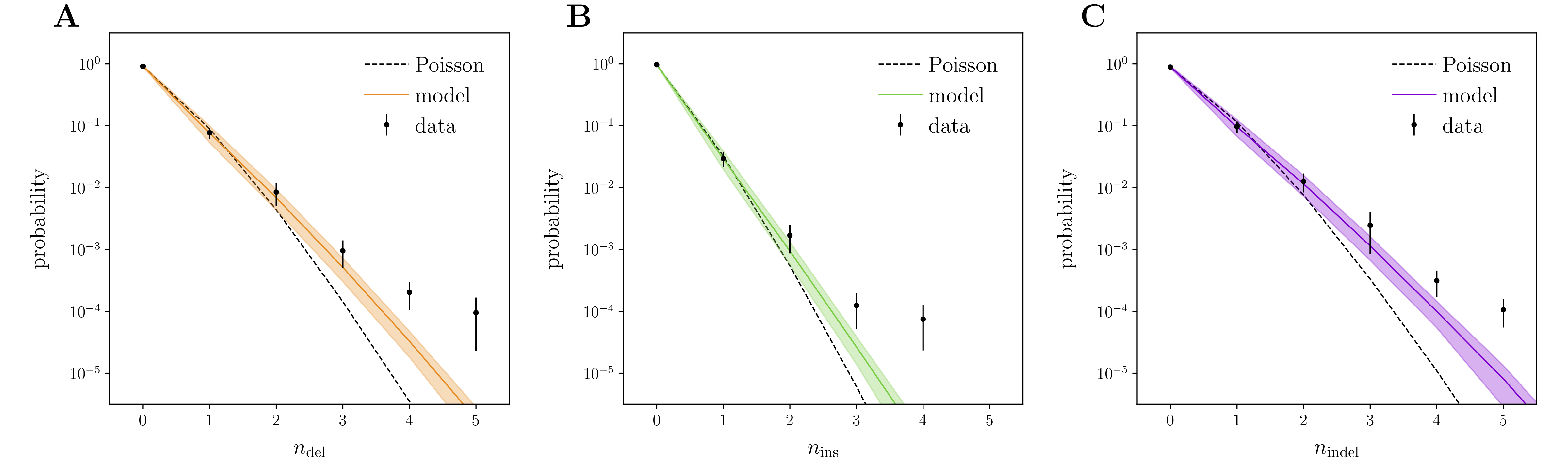

Supplement: S5 Fig — Model prediction for frequency of (A) deletions, (B) insertions, and (C) both insertions and deletions, in IgG nonproductive sequences, compared to the data. Mean and variance are over the 9 donors. Poisson distributions with the same means as the data are shown for comparison. (TIFF) [file pcbi.1010167.s005.tiff]

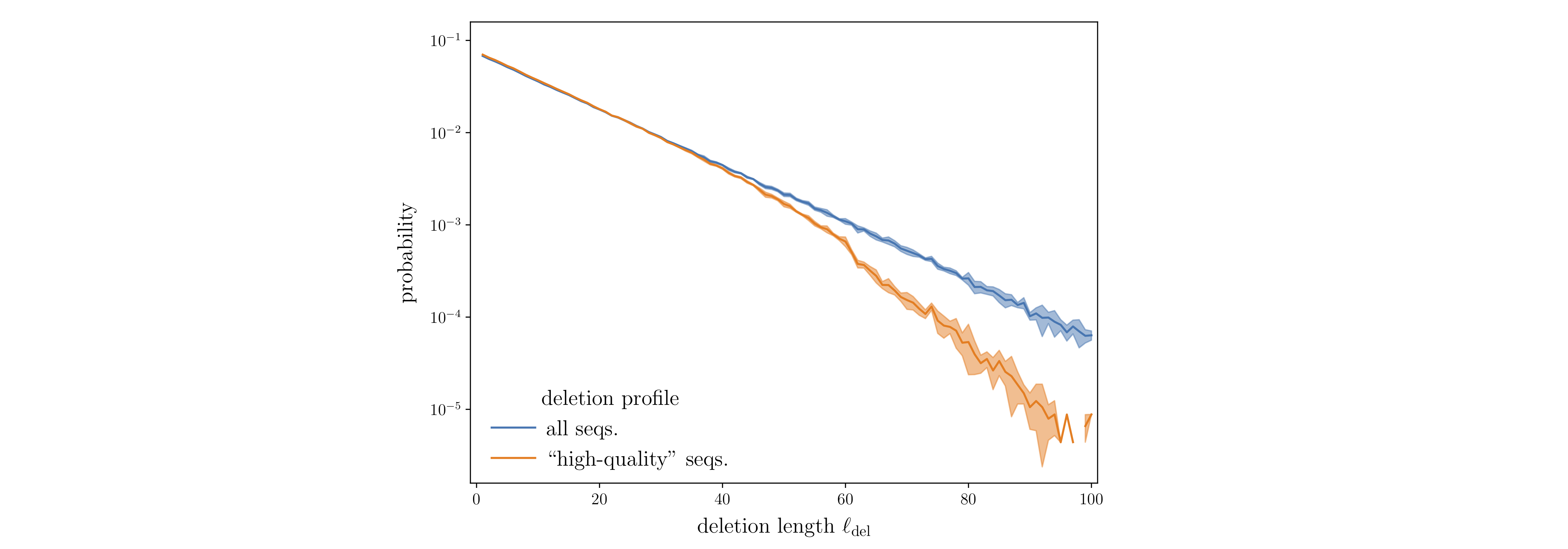

Supplement: S6 Fig — Synthetic sequences were generated as in Fig 3D, but with a cut-off of 100 base pairs instead of 30 for indel lengths, and with indel-to-point-mutation rates βdel = βins = 0.025. The blue curve shows the true distribution of deletions lengths in the full dataset, while the orange curve shows the same distribution in sequences that have passed the quality filters described in Methods section. Mean and standard deviation over the 5 subsets shown. (TIFF) [file pcbi.1010167.s006.tiff]

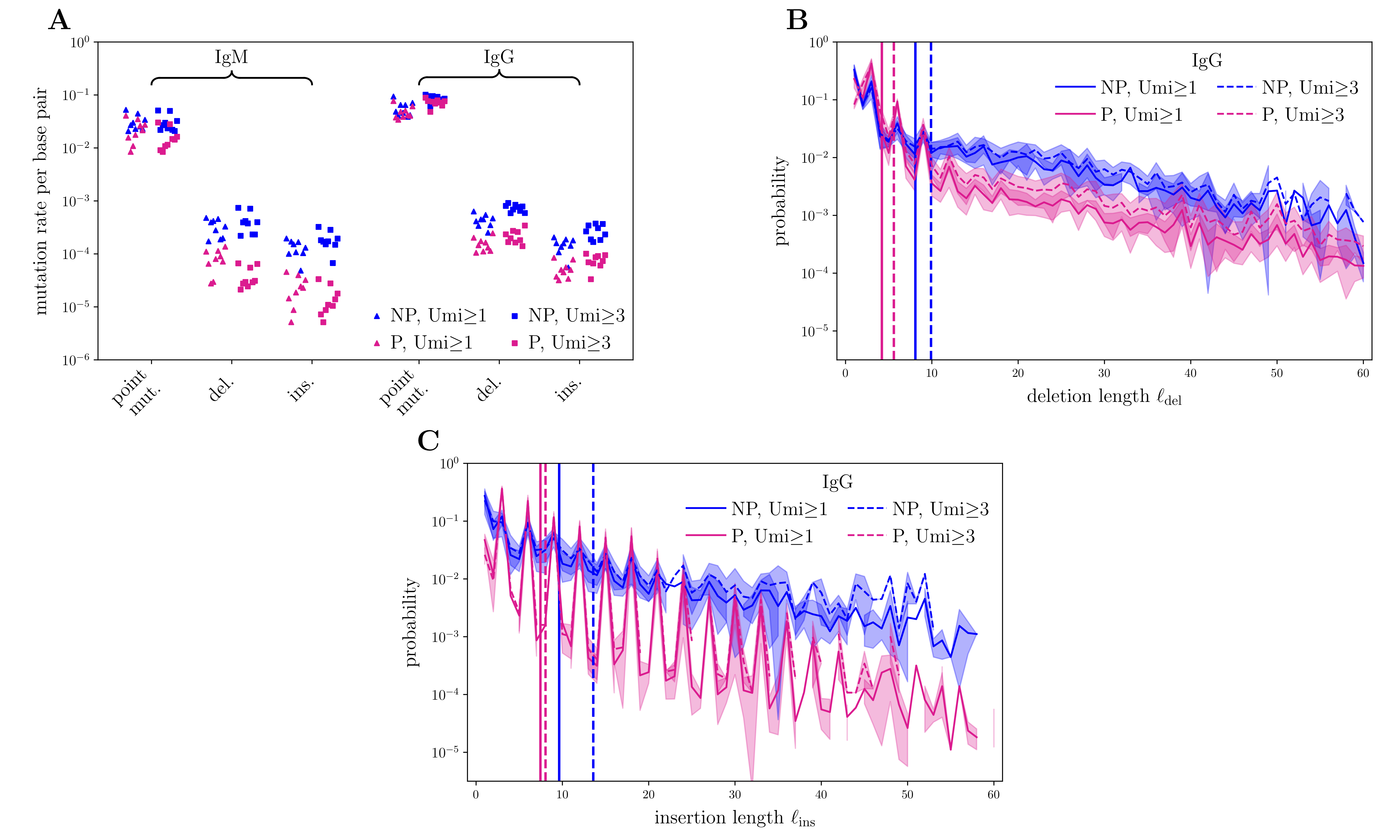

Supplement: S7 Fig — No systematic error due to sequencing errors (expected to be larger in UMI with low counts) can be detected. (A) Mutation rates per base pair, for both IgM and IgG. (B) Length profiles for deletions in IgG. (C) Length profiles for insertions in IgG. Vertical lines show mean values. (TIFF) [file pcbi.1010167.s007.tiff]
